# Improving Urban Stormwater Runoff Quality by Nutrient Removal through Floating Treatment Wetlands and Vegetation Harvest

**DOI:** 10.1038/s41598-017-07439-7

**Published:** 2017-08-01

**Authors:** Bing Xu, Xue Wang, Jia Liu, Jiaqiang Wu, Yongjun Zhao, Weixing Cao

**Affiliations:** 1 School of municipal and environmental engineering, Shandong Jianzhu University, Jinan, 250101 China; 2 Co-innovation center of Green Building, Jinan, 250101 China; 3 Shanghai Public Green Space Construction Affairs Center, Shanghai, 201100 China; 4 Jinan water Group Co, Ltd, Jinan, 250012 China; 5 College of Biological Chemical Science and Engineering, Jiaxing University, Jiaxing, 314001 P.R. China

## Abstract

Two floating treatment wetlands (FTWs) in experimental tanks were compared in terms of their effectiveness on removing nutrients. The results showed that the FTWs were dominated by emergent wetland plants and were constructed to remove nutrients from simulated urban stormwater. *Iris pseudacorus* and *Thalia dealbata* wetland systems were effective in reducing the nutrient. *T*. *dealbata* FTWs showed higher nutrient removal performance than *I. pseudacorus* FTWs. Nitrogen (N) and phosphorous (P) removal rates in water by *T. dealbata* FTWs were 3.95 ± 0.19 and 0.15 ± 0.01 g/m^2^/day, respectively. For *I. pseudacorus* FTWs, the TN and TP removal rates were 3.07 ± 0.15 and 0.14 ± 0.01 g/m^2^/day, respectively. The maximum absolute growth rate for *T*. *dealbata* corresponded directly with the maximum mean nutrient removal efficiency during the 5th stage. At harvest, N and P uptak of *T*. *dealbata* was 23.354 ± 1.366 g and 1.489 ± 0.077 g per plant, respectively, approximate twice as high as by *I. pseudacorus*.

## Introduction

Nutrients in urban stormwater runoff primarily originate from the washoff of impervious surfaces and surrounding soils, as well as atmospheric wet and dry deposition^1^. Excess nutrients in urban stormwater runoff have been identified as a reason of the degradation of surface water quality^2^. The first flush of urban stormwater runoff is highly enriched in nutrients, such as nitrogen (N) and phosphorus (P), resulting in eutrophication of downstream water bodies and impairment of aquatic ecosystems^3, 4^.

To control the quality of urban stormwater and reduce pollutant mass loading prior to discharge into receiving water bodies, floating treatment wetlands (FTWs) as one emerging best management practices (BMPs) which restore receiving water using a variety of biological and physical processes, provide nutrients removal through simultaneously hydrological and biological controls relating to various hydrobiogeochemical processes in a multimedia pond environment^1, 5^. In FTWs, plants are grown on floating mats and the root systems of the species are suspended in the water column rather than rooted in sediments, so FTWs offer great promise for rainfall-driven stormwater treatment applications as they are conducive to settling by reducing turbulence and wave mixing induced by wind and thermal factors^6–8^. These are the main key differences between FTWs and traditional wetland systems where the majority of pollutants are removed through the gravel matrix or sediment rather than the water column^9^. Other potential advantages of FTWs, such as low construction costs, the use of vegetation as a food source for animals, providing a habitat for harvestable fish, no requirement for additional land area and aesthetic value, making FTWs an attractive option for meeting water quality standards through nutrient reduction^10^.

FTWs have only been used for a limited range of applications up to now, such as agricultural wastewater treatment, sewage effluent remediation and stormwater quality improvement^11, 12^. Additionally, in recent years, FTWs have been successfully used to remove nutrients, metals, and glycol from stormwater. However, only a few of studies have focused on stormwater. White and Cousins^13^ applied FTWs planted with *Juncus effusus* and *Canna flaccida* to remediating runoff and showed that *Juncus* plants fixed 28.5 ± 3.4 g N per m^2^ and 1.69 ± 0.2 g P per m^2^, while *Canna* plants fixed 16.8 ± 2.8 g N per m^2^ and 1.05 ± 0.2 g P per m^2^. Although there are several papers showing a reduction of pollutants from the released stormwater due to storage of the constituents in plant tissues and attached microorganisms on the floating mats rather than algae suspended in the water body^14^, few research has been conducted to get into the best management strategies for FTWs.

Plant harvesting act as a management strategy facilitate for removal of nutrients from internal wetland cycling processes. A harvesting strategy involving either the above-ground parts of the plants or whole plant tissues should be developed based on either temporal variation in nutrient content in the harvestable tissues or accumulated nutrient removal from the wastewater. For example, Wang *et al*.^15^ reported that the P mass in the pickerelweed aerial parts was 1.0 and 0.6 mg-P/plant in July and September, respectively. These results showed that manual harvesting of above-ground pickerelweed tissues in July instead of September would double the amount of removed P. However, harvesting the whole pickerelweed plant should be conducted in September as accumulated P removal from water was highest at this time. Understanding the temporal variation of nutrient distribution in plants is especially important for optimizing the harvesting strategy.

Over the past few years, FTWs have been studied with the aim of enhancing the effectiveness of retention ponds and protecting water resources^16^. Previous research has shown that dissolved contaminants present in the urban stormwater runoff could be removed efficiently via plants and microorganisms in FTWs^11, 17^. Several studies have focused on the removal of dissolved metals and nutrients present in urban stormwater runoff from airports, highways and residential areas, while other studies have focused on wetland vegetation screening and the structural design of FTWs^18^. However, the influences of seasonal variation on FTWs, plant harvesting strategy, and the role of microorganisms in nutrient removal have not been well studied.

The objectives of this study are to (1) assess N and P removal by two wetland plants, (2) characterize the mechanisms associated with nutrient removal within the microcosms and (3) evaluate the temporal variation of N and P mass in plants to provide recommendations for harvesting strategies to optimize nutrient removal.

## Results and Discussion

### Nutrient removal efficiency of different FTWs

**Figure 1:**
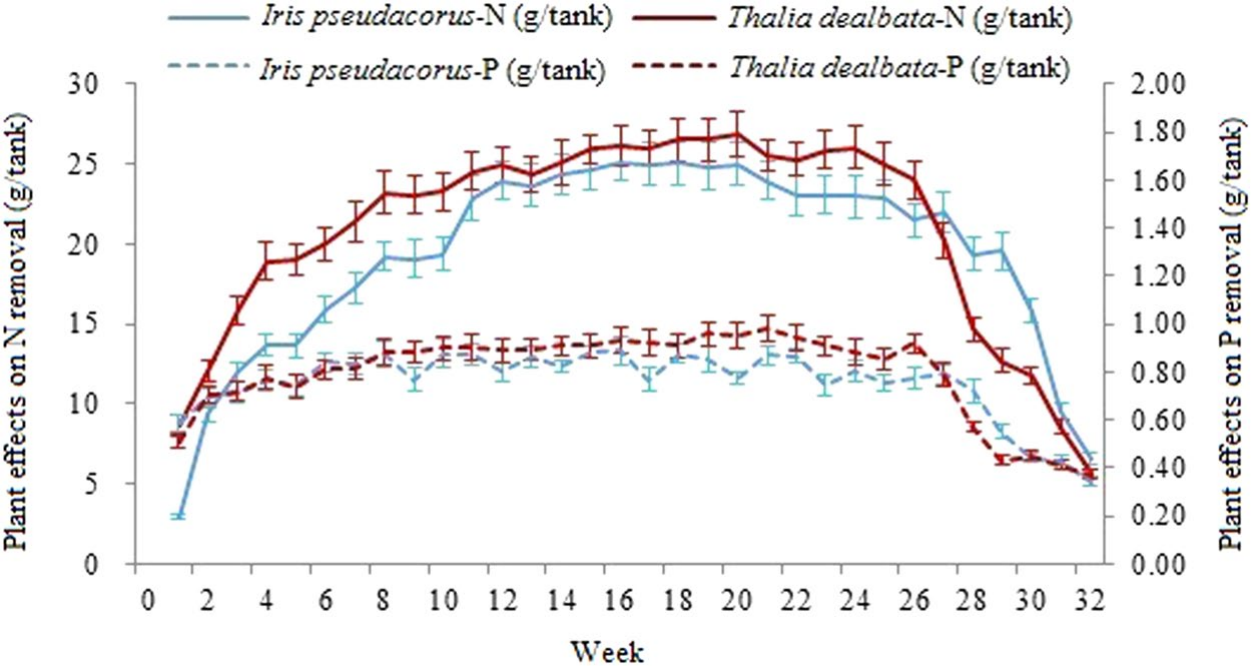
Vegetation effects on mass removal in one experimental tank (mean, n = 3). Each tank contained one FTW with nine plants. Weekly accumulated P and N mass removal in the *Iris pseudacorus* and *Thalia dealbata* FTWs treatment system at each time step were obtained to estimate net contribution of the plant uptake and associated microbial activities in the FTW plants.

There was no significant difference (*p* > 0.05) between *I. pseudacorus* and *T. dealbata* FTWs in terms of pH, dissolved oxygen (DO), TN and TP in the influent water during the operational period (Table 1). However, DO, TN and TP levels in the effluent were significantly lower in *T. dealbata* FTWs than in *I. pseudacorus* FTWs (*p* < 0.05). In this study, differences in average nutrient removal efficiencies (REs) between *T. dealbata* and *I. pseudacorus* FTWs were significant over the study period (*p* < 0.05) (Table 1). The curves in Fig. 1 also show that *T. dealbata* FTWs removed nutrients more effectively than *I. pseudacorus* FTWs in most batches. In other words, the ability to remove TN was significantly different between *I. pseudacorus* and *T. dealbata* over the entire experimental period, except during stages 4 and 5 (July and August) (Fig. 1). However, for TP removal, there was no significant difference between earlier and later batches, excluding the intermediate batches. Average nutrient mass reduction was 22.41 ± 1.13 g for N and 0.83 ± 0.04 g for P by *T. dealbata* (per tank in one batch). For *I. pseudacorus*, the average values were 17.48 ± 0.88 g N and 0.77 ± 0.03 g P per tank in one batch, respectively. Maximum TN mass removal was 26.88 ± 1.42 g and 25.08 ± 1.13 g per tank on August 31 and August 3 by *T. dealbata* and *I. pseudacorus*, respectively (Fig. 1). For TP, the corresponding values were 0.98 ± 0.05 g and 0.89 ± 0.04 g per tank on September 7 and August 3 by *T. dealbata* and *I. pseudacorus*, respectively (Fig. 1). Nutrient mass removal rates per FTW unit area in the simulated urban stormwater runoff were calculated according to the FTW area and the experimental results (Fig. 1). TN and TP mass removal rates by *T. dealbata* FTWs were 3.95 ± 0.19 g/m^2^/day and 0.15 ± 0.01 g/m^2^/day, respectively, while TN and TP removal rates for *I. pseudacorus* were 3.07 ± 0.15 g/m^2^/day and 0.14 ± 0.01 g/m^2^/day, respectively.

**Table 1:** Mean concentration of total nitrogen (TN) and total phosphorus (TP) (mean ± SD, n = 3) of the influent water and effluent water in the FTWs, and mean removal efficiencies/mass in 32 batches.

Wetland types	Influent water				Effluent water				Removal efficiency		Removal mass	
	TN(mg L^−1^)	TP(mg L^−1^)	pH	DO(mg L^−1^)	TN(mg L^−1^)	TP(mg L^−1^)	pH	DO(mg L^−1^)	TN (%)	TP (%)	TN(g/tank)	TP(g/tank)
*I. pseudacorus*	13.25^a^ ± 0.63	0.45^a^ ± 0.02	7.41^a^ ± 0.35	9.89^a^ ± 0.47	4.51^a^ ± 0.24	0.06^a^ ± 0.002	7.45^a^ ± 0.36	8.65^a^ ± 0.41	66.02b ± 3.31	86.59b ± 4.43	17.48b ± 0.86	0.78b ± 0.03
*T. dealbata*	13.41^a^ ± 0.65	0.44^a^ ± 0.01	7.34^a^ ± 0.33	9.91^a^ ± 0.49	2.21b ± 0.13	0.04b ± 0.003	7.43^a^ ± 0.38	8.07b ± 0.42	83.52^a^ ± 3.98	91.01^a^ ± 4.50	22.41^a^ ± 1.12	0.83^a^ ± 0.04

Note: Values with different superscript letters in the same column for the two treatments indicate a significant difference at *P* < 0.05 according to Duncan’s multiple range test.

The mean total N and P removal rates in this study were significantly higher than those found in previous studies^19–21^. For example, Wang *et al*.^22^ reported TN and TP mass removal rates of 0.42 and 0.03 g/m^2^/day by *T. dealbata* in the Hengtanghe River, Yixing, China. It indicated that nutrient removal efficiency of *I. pseudacorus* FTWs improved with increasing of nutrient concentration, and decreased when an N concentration above 80 mg/L^15^. The higher removal rates in this study may be due to the appropriate nutrients concentration used in the water.

The major nutrient removal processes in FTW systems are precipitation, sorption, plant uptake (with subsequent harvest), algae/microbial uptake, and burial^23^. Physicochemical properties of water also impact the performance of FTW. For example, high pH values, which often occur as a result of photosynthesis from algae and submerged macrophytes in wetlands, can enhance nitrogen removal, such as the volatilization of ammonia. There were no significant differences between influent and effluent water pH for the two FTWs in this study(*p* > 0.05). It indicated that the nutrient removal by alage uptake could be neglected. On the other hand, nutrient removal rates are usually affected by nutrient concentrations, hydraulic residence time, temperature and plant species^9^. In this study, the proper nutrient concentration, the form of the nutrients, and the plant species have resulted in more efficient removal of these nutrients compared to that observed in other studies. For example, in terms of nitrogen, different plant species may have a specific tolerance to and preference for NO_3_^−^ or NH_4_^+^, and microbial activity in the plant rhizosphere may be affected by different forms of nitrogen^24^. Under sole NO_3_^−^ treatments, total dry weight (DW), shoot DW, and N content of *I. pseudacorus* were higher than those treated using a mixture of NO_3_^−^ and NH_4_^+^^25^. It can be concluded that species favoring NO_3_^−^, such as *I. pseudacorus* and *T. dealbata*, can be used in FTWs to effectively treat unban stormwater dominated by NO_3_^−^. This result was corresponding to Li’s findings^26^. Over the entire study period, *I. pseudacorus* FTWs twice achieved an average TN mass removal rate above 30.00 g/m^2^(stage 4, 30.15 ± 1.61 g/m^2^,in July and stage 5, 30.20 ± 1.55 g/m^2^, in August). For *T. dealbata* FTWs, three stages achieved above 30.00 g/m^2^ for TN mass removal, namely stage 4 (31.33 ± 1.58 g/m^2^, in July), stage 5 (32.71 ± 1.65 g/m^2^, in August), and stage 6 (31.70 ± 1.60 g/m^2^, in September). Phosphorus removal by *I. pseudacorus* FTWs was also lower than that of *T. dealbata* FTWs during most intermediate stages (Fig. 1).

Previous studies have shown that higher shoot and root biomass production result in better nutrient removal^1, 27^. Wang *et al*.^14^ reported that *Pontederia cordata L*. outperformed *Schoenoplectus tabernaemontani* on P and N removal, based upon higher nutrient accumulation and higher biomass in *P. cordata L*. compared to *S. tabernaemontani*. Overall, it appeared that *T. dealbata* outperformed *I. pseudacorus* until the last two stages (Fig. 1), indicating that *T. dealbata* FTWs are more suitable than *I. pseudacorus* FTWs for urban stormwater runoff treatment.

### Relationship between plant growth, microbial population and nutrient removal

**Figure 2:**
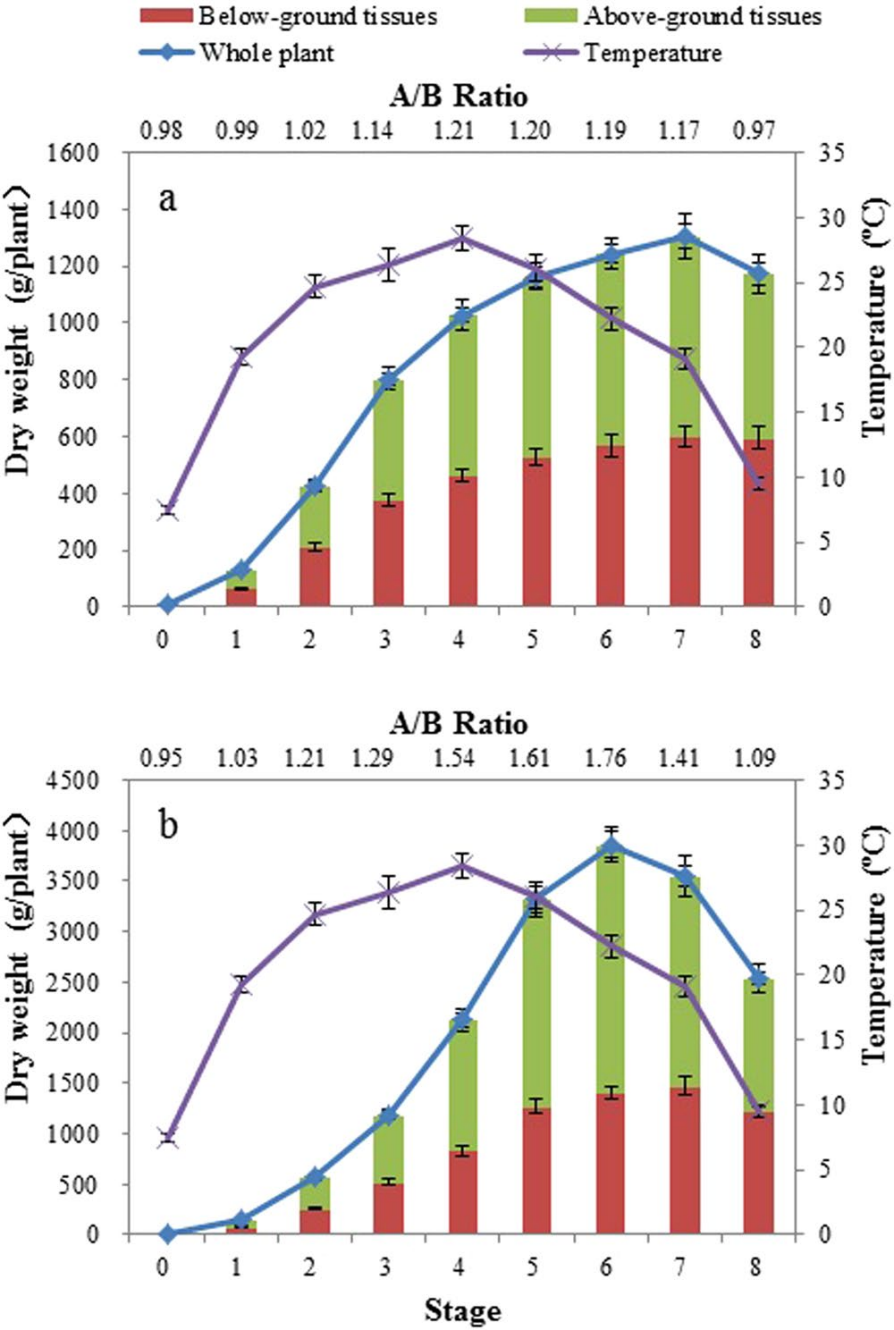
Variations of the plant dry weight of the tested vegetation with time being consistent with the variations of temperature in the eight stages; A/B Ratio was the ratio of above-ground tissue dry weight to below-ground tissue dry weight, (**a**) *Iris pseudacorus*, (**b**) *Thalia dealbata*.

Nutrients can be taken up directly by shoots and roots and be converted into organic compounds that serve as building blocks for cells and tissues^28^. The rate of nutrient removal by FTWs is enslaved to the vegetation net productivity (growth rate), the concentration of nutrients in the plant tissue, and the degree of microbial participation^11, 29^. In this study, the biomass DW of *I*. *pseudacorus* and *T*. *dealbata* reached maximum during stage 7 (October) and stage 6 (September), respectively, after continuous growth during the previous stages (Fig. 2). Growth rate of plant was reduced in the later stages as a result of plant senescence. The data in Table 2 show that the whole plant absolute growth rate (WAGR) of *T*. *dealbata* up to stage 7 was consistently greater than that of *I*. *pseudacorus* in the previous six stages, with a WAGR maximum of 42.63 ± 2.78 g/day at stage 5 (August). Following this, WAGR reduced sharply and was negative during stages 7 and 8 due to tissue withering or death in October and November. In contrast, the WAGR of *I*. *pseudacorus* increased quickly and achieved a maximum value of 13.42 ± 0.77 g/day during stage 3 (June), which then gradually declined to below zero in November due to plant senescence (Table 2).

**Table 2:** Absolute growth rate (mean ± SD, n = 3) in different parts of plant after 28 days mesocosm experiment in each stage.

Stage	*I. pseudacorus*			*T. dealbata*		
	WAGR (g/day)	AAGR (g/day)	BAGR (g/day)	WAGR (g/day)	AAGR (g/day)	BAGR (g/day)
1	4.34 ± 0.24	2.16 ± 0.11	2.18 ± 0.12	5.23 ± 0.27	2.65 ± 0.15	2.58 ± 0.17
2	10.58 ± 0.51	5.38 ± 0.35	5.20 ± 0.31	14.87 ± 0.96	8.36 ± 0.58	6.51 ± 0.40
3	13.42 ± 0.77	7.57 ± 0.34	5.85 ± 0.36	22.18 ± 1.22	12.81 ± 0.83	9.36 ± 0.61
4	8.04 ± 0.41	4.83 ± 0.28	3.21 ± 0.15	33.45 ± 1.67	22.11 ± 1.24	11.35 ± 0.74
5	4.80 ± 0.29	2.54 ± 0.13	2.26 ± 0.14	42.63 ± 2.78	27.10 ± 1.79	15.53 ± 0.96
6	2.91 ± 0.13	1.49 ± 0.09	1.41 ± 0.09	19.36 ± 1.01	14.81 ± 0.77	4.54 ± 0.26
7	2.19 ± 0.13	0.99 ± 0.06	1.20 ± 0.05	−7.61 ± 0.13	−13.85 ± 0.13	2.66 ± 0.16
8	−4.69 ± 0.24	−4.49 ± 0.23	−0.21 ± 0.01	−39.67 ± 2.30	−26.74 ± 1.58	−9.35 ± 0.57

WAGR is the whole plant absolute growth rate, AAGR is the above-ground tissues absolute growth rate and BAGR is below-ground tissues absolute growth rate.

**Figure 3:**
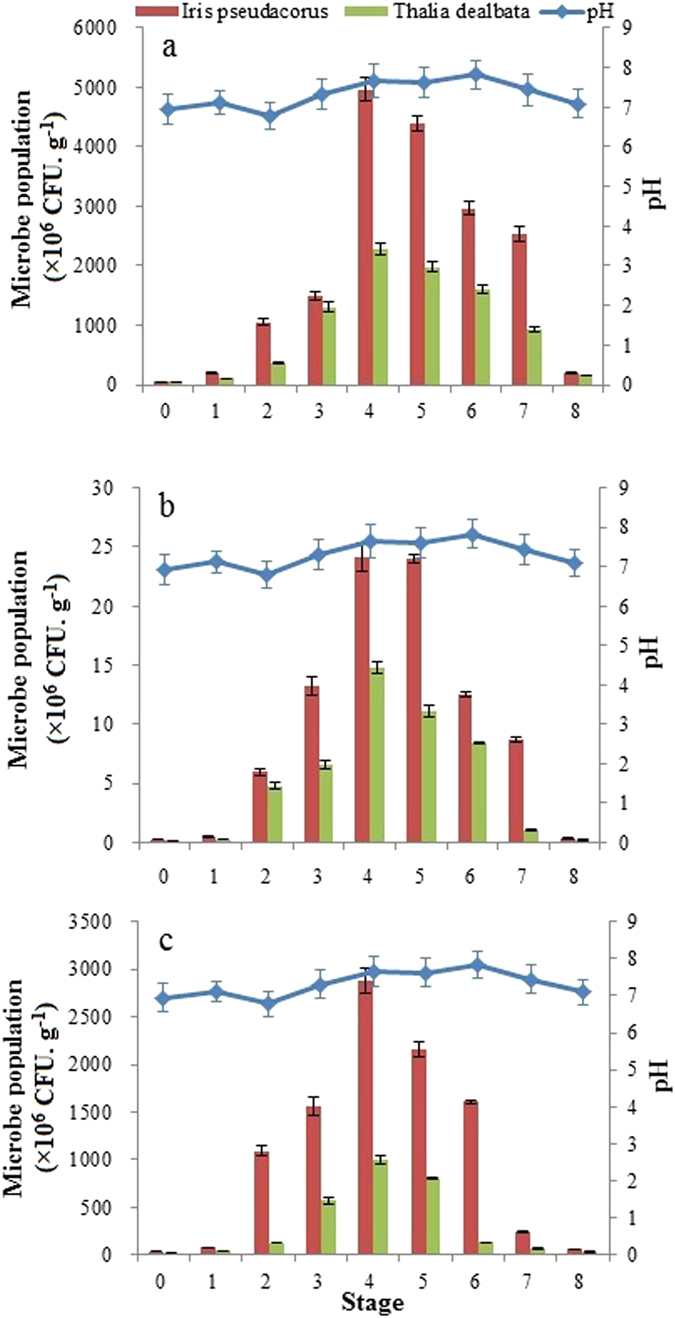
Microbial population in the rhizosphere of floating treatment wetland plants at various stages (×106 CFU∙g^−1^), (**a**) bacteria, (**b**) fungi, (**c**) actinomycetes.

Based on the relationships between temporal variations in AGR or DW of the two plants, combined with variations in nutrient removal (Figs 1 and 2; Table 2), it can be concluded that plant growth plays a significant role in nutrient removal in the FTWs. For example, in *T. dealbata* FTWs, a rapid increase in WAGR from stage 1 (April) to stage 3 (June) corresponded with a sharp increase in nutrient removal between batch 1 to 12 (Fig. 1 and Table 2). It can therefore be confirmed that plant assimilation of nutrients rather than microbial uptake, results in nutrient removal, because the microbial population did not increase as rapidly as the plant root system (Figs 2 and 3; Table 2). These findings have also been observed in studies of a mesocosm treatment wetland system^30^ and a vertical flow constructed wetland^31^. Here, in the previous two stages, the microorganisms in the rhizosphere had developed gradually at a lower productivity rate (Fig. 3). This meant that the microorganisms were in the initialization phase and a stable biofilm had not yet been generated. On the other hand, plant uptake is a spring-summer phenomenon in temperate climates^23^. Both the above- and below-ground parts of *T. dealbata* grow well from April to September, with a maximum growth rate in late summer, together with plant death occurred in later stages (Table 2).

The fluctuation of WAGR and the mature microbial community from stage 4 to 6 may have resulted in nutrient removal fluctuations, which appeared as a wave-like pattern from batch 13 to 24, with a maximum value for TN: 26.88 ± 1.42 g/tank and TP of 0.98 ± 0.05 g/tank) (Fig. 1 and Table 2). However, it was notable that strong variation of WAGR in stages 4, 5, and 6 were not consistent with the small changes in TN removal efficiency in *T. dealbata* FTWs. There are two possible reasons for this result. First, nutrient removal efficiency was limited by the initial nutrient concentration, which was not high enough to maintain plant and microbial productivity. In those stages, the nutrients were almost exhausted based on nutrient removal efficiencies in stage 4 (TN: 95.53 ± 4.87%, TP: 98.71 ± 4.91%), stage 5 (TN: 96.16 ± 4.88%, TP: 99.48 ± 4.95%) and stage 6 (TN: 93.77 ± 4.75%, TP: 98.83 ± 4.98%). Second, microbial uptake played a vital role in nutrient removal in those stages. In particular, the WAGR in the stage 4 was lower than that in stage 5, while the microbial population was relatively high (Table 2 and Fig. 3). This finding may be due to small differences in TN removal efficiencies between stage 4 and 5, where the average percent contribution of *T. dealbata* uptake accounts for 57.94 ± 2.95% and 68.34 ± 4.51%, respectively. Furthermore, the microbial population reduced sharply with a rapid decline in nutrient removal when *T. dealbata* withered during stages 7 and 8.

Nutrient removal by *I. pseudacorus* FTWs was similar to that of *T. dealbata* FTWs. However, there were no significant differences in terms of average TN mass removal between *I. pseudacorus* FTWs and *T. dealbata* FTWs during stages 4 and 5 (*p* > 0.05). Average TN removal efficiencies were more than 94% for *I. pseudacorus* FTWs during those stages. However, the average percent contribution of *I. pseudacorus* for TN mass removal only accounted for 39.49 ± 2.04% and 35.03 ± 1.81%. Compared to those for *T. dealbata* FTWs, it was apparent that there was a non-significant (*p* > 0.05) difference in the average TN removal efficiencies and a significant(*p* < 0.05). difference in the average percent contributions between the two plant species.

The purification performance of FTWs was based on the combined action of microorganisms and plants. Keizer-Vlek *et al*. showed low absolute amounts of TN and TP removed from control tanks (covered with styrofoam)^29^, suggesting that biofilm growth on tank walls and/or styrofoam does not play a major role in TN and TP removal in the presence of plants. In contrast, our results suggested that the majority of nitrogen could be removed by microorganisms both in aerobic and anaerobic conditions in the middle stages, especially in *I. pseudacorus* FTWs. This finding confirmed the role of the microbial biofilms in the rhizosphere, which could also be verified by the microbial population data (Fig. 3). Here, the population of bacteria (Fig. 3a), fungi (Fig. 3b), and actinomycetes (Fig. 3c) in the two FTWs gradually increased and reached maximum values at stage 4, before declining during the later stages. It was apparent that TN removal by microorganisms may compensate for a lack of plant uptake in the middle stages, as the rank of the three stages, according to the three microbial populations, was stage 4 >stage 5 >stage 6.

Previous study found that the microbial community structure was affected by plant species rather than plant richness^32^. On the one hand, microbial biomass can also be strengthened by root productivity. Plant root exudates maintain high microbial diversity and activity^33^. Exudation patterns undergo changes with plant age and location along the root system. Temporal variations in microorganisms (Fig. 3) in this study implied that exudates of *I. pseudacorus* might be more beneficial for the growth of microorganisms than those of *T. dealbata*, possibly resulting in better nutrient removal by *I. pseudacorus* FTWs in later stages. On the other hand, plant roots can enhance oxygen conditions and support aerobic processes in the rhizosphere^33^. In this study, *I. pseudacorus* roots were thicker than those of *T. dealbata*. A thick network of roots provided a larger surface area for biofilm development, and associated biofilms contain communities of attached microorganisms responsible for a number of important treatment processes. In this study, the pH ranged from 7.34 to 7.45, which did not impede microbial activities. Paredes *et al*.^34^ also found that the optimal pH for the growth of groups of bacteria that play a key role in the nitrification, denitrification and ammoniation, was between 7.0 and 8.0.

### Plant harvesting strategies for nutrient removal

In terms of management strategies, nutrients in plant tissues can be removed from urban stormwater ponds through harvesting. The optimal harvesting strategy is based on the nutrient content in the plant tissues, cost-effectiveness, and operational complexity. As shown in Table 3, plant N and P content and distribution in the above-ground and below-ground tissues varied with the seasons, which directly affected the efficiency of nutrient removal through harvesting. Only variations in the nutrient content in below-ground tissues of *I. pseudacorus* and above-ground tissues of *T. dealbata* were consistent with variations in whole plant DW (Table 3 and Fig. 2). In addition, both plant species had their maximum nutrient content on October 26 (stage 7). These results indicated that nutrients in the aerial parts may translocate into the subaqueous fraction of the plant. Vymazal^23^ pointed out that translocation of nutrients within the plant is an important response to seasonal variation. Wang *et al*.^14^ also reported that *P. cordata L*. translocated most resources from the above-ground tissues to below-ground storage organs in autumn. Prior to the senescence phase, the majority of important ions are transported from the shoots to the roots and rhizomes and are used during early spring growth^23^.

**Table 3:** Nutrient mass (mean ± SD, n = 3) in single plant above/below ground tissues after 28 days mesocosm experiment in each stage.

stage	Date	Nitrogen (g-N/plant)				Phosphorus (g-P/plant)			
		plant above ground tissues		plant below ground tissues		plant above ground tissues		plant below ground tissues	
		*I. seudacorus*	*T. dealbata*	*I. pseudacorus*	*T. dealbata*	*I. pseudacorus*	*T. ealbata*	*I. pseudacorus*	*T. dealbata*
0(initial)	4/13	0.105 ± 0.005	0.068 ± 0.003	0.086 ± 0.005	0.032 ± 0.001	0.033 ± 0.002	0.037 ± 0.002	0.030 ± 0.001	0.022 ± 0.001
1	5/11	0.881 ± 0.046	1.947 ± 0.096	0.721 ± 0.041	1.315 ± 0.055	0.101 ± 0.005	0.176 ± 0.010	0.094 ± 0.006	0.086 ± 0.005
2	6/8	2.664 ± 0.135	4.534 ± 0.229	2.180 ± 0.105	2.707 ± 0.199	0.227 ± 0.014	0.395 ± 0.021	0.209 ± 0.009	0.181 ± 0.009
3	7/6	5.695 ± 0.262	8.726 ± 0.446	4.852 ± 0.217	3.925 ± 0.235	0.462 ± 0.022	0.630 ± 0.033	0.445 ± 0.020	0.304 ± 0.019
4	8/3	8.165 ± 0.405	12.927 ± 0.695	6.679 ± 0.304	6.259 ± 0.312	0.575 ± 0.031	0.875 ± 0.041	0.578 ± 0.029	0.432 ± 0.026
5	8/31	10.297 ± 0.511	18.481 ± 1.075	8.356 ± 0.469	8.752 ± 0.492	0.683 ± 0.033	1.221 ± 0.055	0.621 ± 0.034	0.621 ± 0.031
6	9/28	12.258 ± 0.641	23.354 ± 1.366	9.624 ± 0.485	10.026 ± 0.556	0.781 ± 0.041	1.489 ± 0.077	0.728 ± 0.038	0.762 ± 0.039
7	10/26	12.154 ± 0.630	21.513 ± 1.368	10.842 ± 0.551	10.415 ± 0.631	0.807 ± 0.046	1.397 ± 0.075	0.922 ± 0.045	0.894 ± 0.048
8	11/23	11.109 ± 0.623	18.185 ± 1.351	10.527 ± 0.515	9.111 ± 0.618	0.756 ± 0.035	1.255 ± 0.065	0.891 ± 0.051	0.871 ± 0.044

Nutrient storage and translocation from above- to below-ground tissues is affected by the plant species and the external environment. Ideally, harvesting should occur during the maximal nutrient mass storage phase before nutrients were released back from the biomass to the wetland ecosystem due to plant decay. In the current study, maximal N and P mass storage in *T. dealbata* above-ground tissues and maximum N mass storage in *I. pseudacorus* above-ground tissues occurred on September 28 (stage 6); other maximum were reached on October 26 (stage 7) (Table 3). Those of *T. dealbata* corresponded to a maximum value ratio between the above-ground DW and below-ground DW (A/B ratio: 1.76 showed Fig. 2) at the same stage. Other results in terms of death of the above-ground and growth of below-ground tissues of *T. dealbata* had resulted in the variations in nutrient content in the later stages (Table 2 and Table 3). In regard to nutrient content in the above-ground plant tissues, those of *T. dealbata* were higher than those of *I. pseudacorus*, and the maxima were approximately twice as high at stage 6 (Table 3).

Based on our results, harvesting of the above-ground plant tissues was the best practice due to the low cost and simple operation, in addition to taking account of the nutrient distribution in plant tissues. Costs will be further reduced if the roots can overwinter, as the reserved roots can then be used for vegetation restoration in the following spring. Ruiz and Velasco^35^ also stated that the nutrients stored in the below-ground tissues (i.e. rhizomes) of *P. australis* were needed for reproduction in the subsequent growth period. In the current study, the roots of *T. dealbata* and *I. pseudacorus* were safe from fatal damage below zero degrees and develop the following year. Hoffmann *et al*.^36^ reported that above-ground tissue harvesting was more protective, easier and more sustainable than whole plant harvesting. Whole plant harvesting is also problematic as it is difficult to separate the plant from the FTW matrix and completely removes the attached microorganisms.

**Figure 4:**
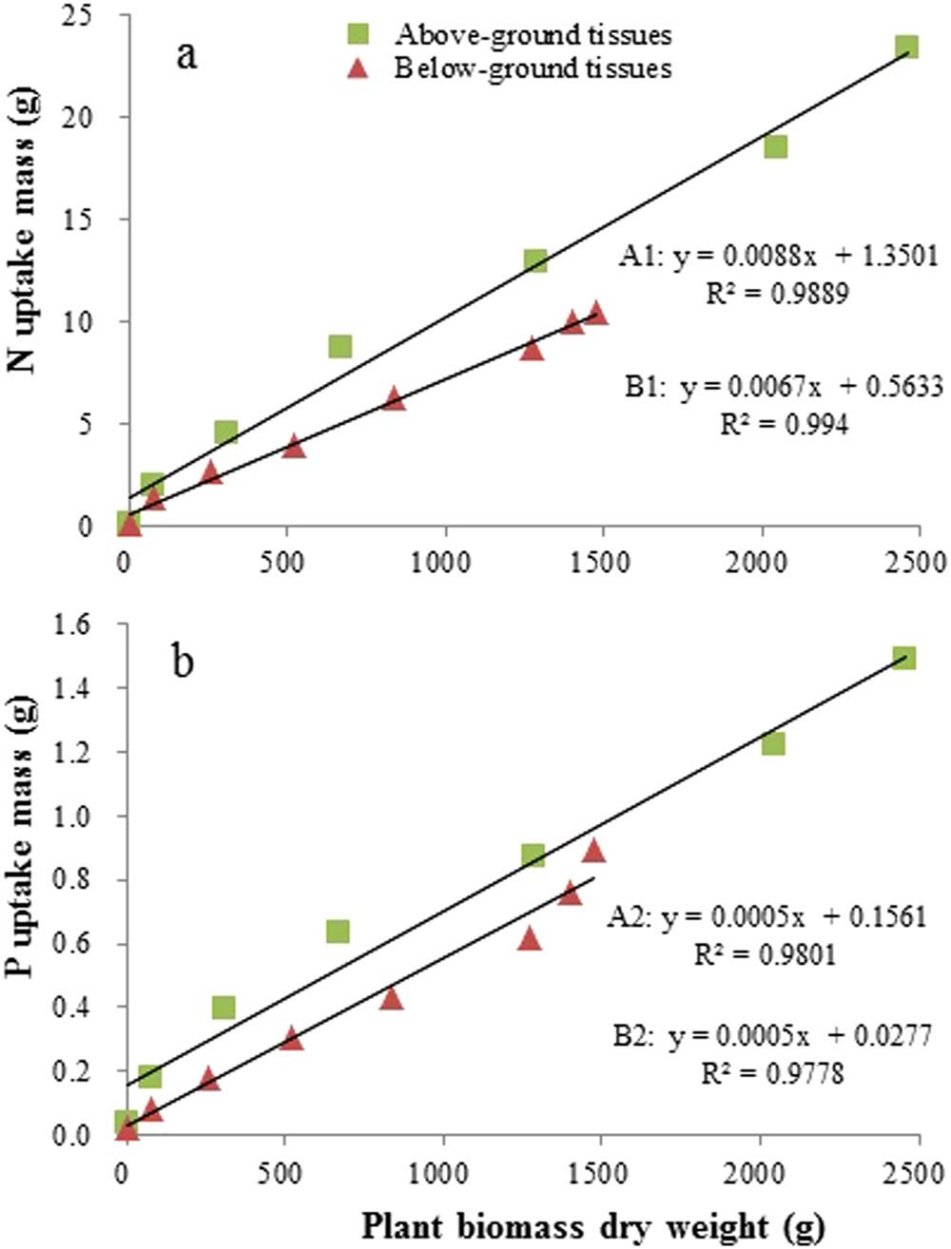
Correlation between nutrients uptake by *T. dealbata* and plant biomass dry weight, (**a**) N uptake mass, (**b**) P uptake mass, (A1) equation between N and above ground tissues, (A2) equation between P and above ground tissues, (B1) equation between N and below ground tissues, (B2) equation between P and below ground tissues.

Given the results from the biomass measurements, the above-ground plant tissues of *T. dealbata* should be harvested in late September (stage 6). The nutrient mass in the above- and below-ground tissues of the plants could be assessed depending on the linear correlation equation (Fig. 4). In terms of N and P uptake by *T. dealbata*, the correlation between the mass of accumulated nutrients and plant biomass DW was significant. In particular, the slope coefficients and intercepts of equations A1 and A2 were greater than those of equations B1 and B2 (Fig. 4).These results suggested that differences in nutrient uptake mass were a result of the different of biomass, and indicated that the harvest of above-ground plant tissues was the optimal strategy.

## Conclusion

This study investigated the performance of two plant species in terms of nutrient removal in experiments using commercially available FTW technologies for urban stormwater runoff treatment. *T. dealbata* showed a higher ability to remove nutrients than *I. pseudacorus*. The microorganisms in the rhizosphere played a significant role in the nutrient removal process and compensated for the lack of plant uptake in the high efficiency removal stages. A harvesting strategy involving the removal of above-ground plant tissues of *T. dealbata* in late September was recommended.

## Materials and Method

### Study site

The experimental site was located near the main campus of Shandong University in the City of Jinan (36°39′N, 117°06′E). The potential of FTWs for urban stormwater runoff quality improvement was studied in lab-scale. Urban stormwater runoff from a heavy traffic road, an airport, and a train station in Jinan were investigated. From 2010 to 2015, average annual precipitation of the catchment ranged from 612–736 mm, with average monthly temperatures ranging from 7.5 to 28.4 °C in April and August, respectively.

### Experimental setup and design

**Figure 5:**
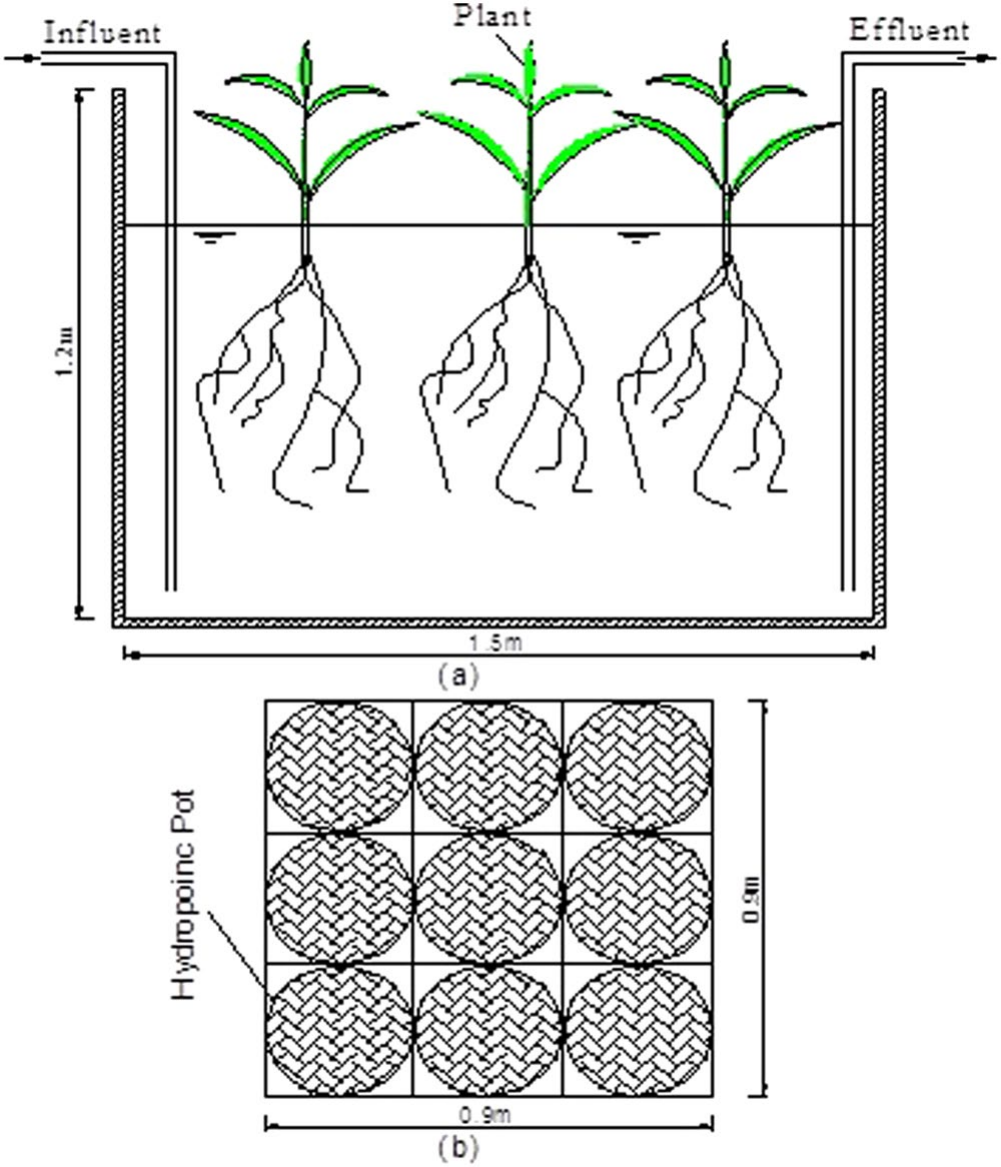
Schematic representation of the experimental system: (**a**) tank and (**b**) constructed floating bed unit.

The FTW experiments were conducted from April 13 to November 23, 2015 and this period was divided into eight stages, with each stage comprising 28 days. There were four batches in one stage and seven days in one batch. The water in the experiment was simulated urban stormwater runoff and was stored in a large pond (5 × 3 × 2.0 m, *L* × *W* × *D*) before being supplied to the FTWs. A complete description of the experimental makeup of the water is available in section of water sampling and analysis. Twelve polyvinyl chloride experimental tanks (1.5 × 1.5 × 1.2 m, *L* × *W* × *D*) were installed under a clear horticultural plastic shelter to facilitate photosynthesis and to eliminate the influence of strong winds, rainfall, bird droppings, and falling leaves^11^. The water surface area of each tank was 2.25 m^2^ with approximately 0.89 m operational water depth, with the inlet and outlet at the bottom of each tank. A detailed diagram of the tanks is shown in Fig. 5. The twelve floating beds (0.90 × 0.90 × 0.15 m, *L* × *W* × *D*) were made of pot holders, polyvinyl chloride pipes, and plastic mesh. There were six floating beds for each treatment, with three replicates for each. Plants growing in the other floating beds were used to replace the plants that were removed for sampling in the two treatments. A single floating bed covered a total of approximately 36% of the water surface area of each tank. Each floating bed had nine ring-shaped pot holders with a 13.8 cm diameter for the hydroponic pots (Fig. 5). Experimental plants (*I. pseudacorus* and *T. dealbata*), 25–30 cm in height (Nanjing Botanical Garden, Nanjing, China), were chosen based upon previous studies that evaluated the adaptability of the roots of these species for long-term submergence in wetland conditions^1^. Plants were transplanted into the 14.5 cm top diameter hydroponic pots after flushing with tap water and were fixed with bristle coir fiber, which provides sufficient porosity and substrate for microbial colonization. The hydroponic pots were numbered and put into the pot holder on the floating beds. After a growth period of 28 days at each stage, selected plants were harvested and their DWs were measured.

To assess the potential of *I. pseudacorus* and *T. dealbata* FTWs for urban stormwater runoff treatment in Jinan, a two factor experiment was conducted with three replicates (Table 4). All tanks were operated in parallel.

**Table 4:** Growing state of the tested plants (mean ± SD, n = 3) in current study.

Plant species	Survival rate(%)	Initial density (plant m^−2^)	Tiller number per plant (plant/plant)	Replicates (tank)
*I. pseudacorus*	99.75 ± 4.82	11	37 ± 1	3
*T. dealbata*	98.69 ± 3.90	11	79 ± 2	3

### Water sampling and analysis

Differences in the characteristics of the urban stormwater runoff as a result of rainfall, traffic patterns, land-use and maintenance made it necessary to collect five urban stormwater runoff samples after the impervious surface had been washed for 30 min at the pipe outlet in a catchment with an area of 1.5 km^2^ at each of three sites: Jiefang road, Jinan railway station and Jinan airport in 2014. Experimental water was simulated based upon the analysis of this collected urban stormwater runoff from 2014. Local data indicated that total nitrogen (TN) concentration ranged from 9.51 to 16.86 mg/L and total phosphorus (TP) concentration ranged from 0.22 to 0.65 mg/L, while mean concentrations of the nutrients of the samples from 15 stormwater events were 13.35 ± 0.64 mg/L for TN and 0.44 ± 0.02 mg/L for TP, respectively. In the current study, nitrate (NO_3_^−^) and phosphate (PO_4_^−^) were used as indicators for models of nitrogen and phosphorus loading. The experimental water was modified in a large pond with KNO_3_, KH_2_PO_4_, and freshwater. The physicochemical characteristics and the mean concentrations of the nutrients of the experimental water during the eight stages are shown in Table 1.

According to the batch treatment operational process, 2 m^3^ of fresh experimental water in the large pond was synchronously injected into the 12 tanks at the start of each batch (8:30 a.m. on the first day) after the previous experimental water in the tanks was completely emptied (8:00 a.m. on the seventh day). During each batch, the inflow and outflow of the water was performed smoothly and slowly to avoid disturbing the biofilm and the microorganisms in the rhizosphere. To alleviate potentially inconsistent evaporation effects between treatments, reverse osmosis (RO) water was added to each tank to the original water level on day 3 and 6 in each batch. A total of 500 mL of the influent and effluent water was sampled on day 0 and 7 in each tank. The analyzed indexes included soluble TN, soluble TP, pH, DO, and temperature (T). The variables pH, DO, and T were measured in the field. The pH and T were recorded using a portable pH meter equipped with microprobes (HACH, HQ30d, pHC28101 electrode, USA) and DO was determined using a portable hand-held dissolved oxygen meter (HACH, HQ30d, LDO10101 electrode, USA). Water samples were filtered through a 0.45 μm glass microfiber filter (GF/C, Whatman, USA) to measure soluble TN and soluble TP concentrations. TN was analysed by persulfate digestion ultraviolet spectrophotometry and TP was analyzed by potassium persulfate digestion MoeSb anti-spectrophotometry^37^. Prior to sampling and analysis, all laboratory glassware was acid-rinsed and flushed with deionized water.

### Plant sampling and analysis

At the initial (0) stage, plant roots were brushed gently with a banister brush and flushed carefully with tap water, in order to remove any loose biofilms, before being transferred to the hydroponic pots. Plants removed for sampling from each treatment tank after 28 days (at the end of one stage) were replaced by those in the pot with the same number in the other floating beds. One whole plant was sampled at 7:30 a.m. on day 28 during one stage. Samples were washed gently and brushed carefully to eliminate any adherent external material, and were wrapped in absorbent paper. During this process, the shoots and roots of the plants were carefully conducted to avoid damage. Subsequently, the plants were cut into two parts: above ground (leaves and stems) and below ground (roots and rhizomes). The above/below ground tissues were sliced into small pieces and mixed well. Biomass DW was determined by exsiccation in a fan-blown electric drying oven (BOXUN, Model: GZX-9246MBE) at 80 °C. The above/below ground tissues were ground using a high-speed grinder (Wiley Mill, Thomas Scientific, Model: 3379-K38) and the obtained powder was passed through a 35-mesh (0.5 mm) sieve. P content in plant tissue was determined after wet oxidation of the dried and ground sample. N content in plant tissue was analyzed by combustion of 30 mg of finely ground sample using an Elemental Analyzer (Thermo Fisher Scientific Inc., Model: EA 1112, Flash 2000)^11^.

### Microorganism sampling and analysis

At the end of each stage, five rhizosphere samples with their attached biofilms (n = 3 for each species) were collected and mixed into a composite sample in sterile glass bottles. The attached biofilm was removed by shaking the samples at 220 rpm for 3 h and the obtained microbial samples were centrifuged twice at 1,000 rpm for 20 min under aseptic conditions, then stored at 4 °C prior to the analysis of microbial parameters (community composition). After cultivation at 28 °C for 48 h, the microbial population was counted using the dilution-plate method. The culture medium for aerobic bacteria, fungi, and actinomycetes were beef extract protein agar, potato-martin substratum, and NA, respectively.

### Calculation and statistical analysis

The removal efficiency (*E*, %) of each treatment in each batch (seven days) was calculated based on Eq. (1):

1 $$E( \% )=(1-\frac{{C}_{f}}{{C}_{i}})\times 100$$

where *C*_i/f_ (mg/L) is the constituent concentration of the influent water/effluent water in each batch.

Average nutrient removal rates (*R*, g/m^2^-day) over the study period (224 days) were calculated based on Eq. (2):

2 $$R=\frac{\sum _{i=0}^{n}{C}_{i}{V}_{i}{E}_{i}}{nAHRT}$$

where *C*_i_ (mg/L) is the nutrient concentration, *V*_i_ (L) is the volume of the influent water in i batch, *E*_i_ is the removal efficiency in batch i, *n* is the batch number, *A* (m^2^) is the FTW area, and *HRT* is the hydraulic retention time (d).

Plant absolute growth rate (*AGR*) was calculated according to the DW changes of the plant at each stage. The calculation of the *AGR* is shown in Eq. (3):

3 $$AG{R}_{i,j,\delta }=\frac{D{W}_{i,j,\delta ,{t}_{2}}-D{W}_{i,j,\delta ,{t}_{1}}}{{t}_{2}-{t}_{1}}$$

where *AGR*_j,j,δ_ is the average relative growth rate at stage i, group j (g·day^−1^), *DW* is the dry weight (g), t_1_ is the initial time of stage i (0 days), t_2_ is the initial time of stage i (28 days), and δ is the tissue type (whole plant, above-ground tissue, or below-ground tissue).

In this study, each sample was tested in triplicate. Analysis of variance (ANOVA) was performed using the general linear model in the univariate procedure with SPSS software (SPSS 15.0, SPSS Inc., Chicago, IL, USA). Values are expressed as means ± standard error (S.E.). One-way analysis of variance was conducted with a significance level of *p* < 0.05.
